# Bilirubin Nanoparticle Treatment in Obese Mice Inhibits Hepatic Ceramide Production and Remodels Liver Fat Content

**DOI:** 10.3390/metabo13020215

**Published:** 2023-02-01

**Authors:** Zachary A. Kipp, Genesee J. Martinez, Evelyn A. Bates, Agil B. Maharramov, Robert M. Flight, Hunter N. B. Moseley, Andrew J. Morris, David E. Stec, Terry D. Hinds

**Affiliations:** 1 Department of Pharmacology and Nutritional Sciences, University of Kentucky, 760 Press Avenue, Healthy Kentucky Research Building, Lexington, KY 40508, USA; 2 Department of Molecular and Cellular Biochemistry, University of Kentucky, Lexington, KY 40508, USA; 3 Markey Cancer Center, University of Kentucky, Lexington, KY 40508, USA; 4 Institute for Biomedical Informatics, University of Kentucky, Lexington, KY 40508, USA; 5 Center for Clinical and Translational Sciences, University of Kentucky, Lexington, KY 40508, USA; 6 Department of Pharmacology and Toxicology, University of Arkansas for Medical Sciences, Little Rock, AR 72205, USA; 7 Department of Physiology & Biophysics, Cardiorenal, and Metabolic Diseases Research Center, University of Mississippi Medical Center, Jackson, MS 39216, USA; 8 Barnstable Brown Diabetes Center, University of Kentucky, Lexington, KY 40508, USA

**Keywords:** bilirubin, ceramides, NAFLD, lipidomics, HO-1, Blvra, heme oxygenase, lipids, hepatic steatosis, obese

## Abstract

Studies have indicated that increasing plasma bilirubin levels might be useful for preventing and treating hepatic lipid accumulation that occurs with metabolic diseases such as obesity and diabetes. We have previously demonstrated that mice with hyperbilirubinemia had significantly less lipid accumulation in a diet-induced non-alcoholic fatty liver disease (NAFLD) model. However, bilirubin’s effects on individual lipid species are currently unknown. Therefore, we used liquid chromatography-mass spectroscopy (LC-MS) to determine the hepatic lipid composition of obese mice with NAFLD treated with bilirubin nanoparticles or vehicle control. We placed the mice on a high-fat diet (HFD) for 24 weeks and then treated them with bilirubin nanoparticles or vehicle control for 4 weeks while maintaining the HFD. Bilirubin nanoparticles suppressed hepatic fat content overall. After analyzing the lipidomics data, we determined that bilirubin inhibited the accumulation of ceramides in the liver. The bilirubin nanoparticles significantly lowered the hepatic expression of two essential enzymes that regulate ceramide production, *Sgpl1* and *Degs1*. Our results demonstrate that the bilirubin nanoparticles improve hepatic fat content by reducing ceramide production, remodeling the liver fat content, and improving overall metabolic health.

## 1. Introduction

The prevalence of non-alcoholic fatty liver disease (NAFLD) is on the rise worldwide, increasing from 25.5% in 2005 to an estimated 32.4% in 2022 [1]. NAFLD is the most common liver disease, which is characterized by an increase in lipid accumulation (hepatic steatosis) and inflammation within the liver [2,3]. NAFLD can cause the development of comorbidities, including diabetes and cardiovascular disease [4,5]. Even with such a high prevalence of NAFLD, no approved pharmacological therapies exist. Recent studies have investigated the therapeutic potential of bilirubin [6]. Bilirubin has been shown to directly bind to the nuclear receptor peroxisome proliferator-activated receptor alpha (PPARα) and activate its transcriptional activity [7,8,9,10]. This activation has been demonstrated to improve metabolic dysfunction in rodents with diet-induced NAFLD and its related comorbidities [10,11]. Within the adipose tissue, bilirubin has been shown to reshape the coregulator profile of PPARα, increase the number of mitochondria, and decrease the adipocyte size [10]. In the liver, bilirubin lowers lipid accumulation by inhibiting de novo lipogenesis and activating the hepatic β-oxidation pathway [11].

This study aimed to determine if bilirubin nanoparticles change the lipid species within the liver, which might protect against diet-induced NAFLD. This was achieved using a model of chronic high-fat diet feeding in mice as they developed NAFLD. The specific lipid species that accumulate within the liver during NAFLD are critical for determining the prognosis and progression of the disease. Ceramides have deleterious effects on the liver and have been linked to the development of insulin resistance and inflammation [12]. There are three distinct mechanisms through which ceramides are produced in the liver. Most commonly, ceramides are formed through de novo ceramide synthesis in the endoplasmic reticulum [13]. Ceramides can also be produced from the hydrolysis of sphingomyelin or through the salvage pathways [14]. For these reasons, serum and liver levels of ceramides have been correlated with the severity of NAFLD in humans [15]. Since inhibiting the formation of ceramides blocks lipid accumulation in rats with NAFLD [16]. Here, we wanted to determine whether bilirubin nanoparticles remodel the lipid species in the liver of mice with NAFLD.

## 2. Materials and Methods

### 2.1. Animals

The experimental procedures and protocols of this study conformed to the National Institutes of Health Guide for the Care and Use of Laboratory Animals. The Institutional Animal Care and Use Committee of the University of Mississippi Medical Center approved them. Eight-week-old male C57BL/6J mice were purchased from Jackson Labs (Bar Harbor, ME, USA) and placed on a 60% high-fat diet (diet #D12492, Research Diets, Inc., New Brunswick, NJ, USA) for 24 weeks with full access to tap water. After this time, mice were randomly assigned to either a treatment group consisting of pegylated bilirubin nanoparticles (PEG-BR) (30 mg/kg every other day, i.p.) (*n* = 6) or vehicle/saline (VEH) (*n* = 5) for 4 weeks while continuing on the high-fat diet as previously described [10,11]. At the end of the 4 week treatment period, mice were euthanized by isoflurane inhalation, and tissues were harvested, weighed, and stored at −80 °C. The livers were sliced and prepared for hematoxylin and eosin (H&E) staining, as we have previously described [11,17,18,19,20,21].

### 2.2. Bilirubin Nanoparticle Synthesis

As we have previously described in [10,11], the synthesis of pegylated bilirubin (PEG-BR) was performed at the Research Institute of Pharmaceutical Sciences at the University of Mississippi (Oxford, MS, USA). In brief, the preparation of PEG-BR was from bilirubin-IX-alpha (Frontier Scientific, Logan, UT, USA) and mPEG2000-NH2 (Sigma-Aldrich, St. Louis, MO, USA), as previously described [17,22,23]. The size and morphology of PEG-BR were determined by transmission electron microscopy (TEM) using a model JEM-2100 (JEOL Ltd., Tokyo, Japan). The purity of the PEG-BR was found to be 95%. For these studies, the PEG-BR was resuspended in saline with slight sonication to dissolve and stored at −20 °C in the dark.

### 2.3. Lipidomics

Lipids were extracted using acidified organic solvents as described previously [24,25,26]. The SPLASH^®^ LIPIDOMIX^®^ Mass Spec lipid class-specific internal standards (Avanti Polar Lipids, Alabaster, AL, USA) were added at the start of the extraction. These are stable isotope-labeled standards for all the major lipid classes with unique masses that do not overlap significantly with the naturally occurring lipids in the samples so that they can be quantified independently. Dried lipid residues were reconstituted for analysis. The instrument system was a Shimadzu Nexera UHPLC system coupled with an AB Sciex 6500+ Q-Trap linear ion trap/triple quadrupole mass spectrometer. Lipids were separated by HILIC chromatography using a Phenomenex Luna Silica Column with a guard column of the same material and detected in multiple reaction monitoring modes using method adaptations in the literature. In brief, this method takes advantage of the ability of HILIC chromatography to separate the major classes of glycerolipids and sphingolipids, which then enables the use of time-scheduled measurements of multiple lipid species within each class. Weakly alkaline solvents (pH 8.0) enhance the detection of anionic lipids in negative-ionization mode. Most lipids were monitored as their precursor molecular ions, but in some instances, lipids were monitored as ammoniated adducts. The high sensitivity and speed of the instrument allow for the accurate integration of chromatographic peaks. The method was optimized using a standard mouse liver lipid extract to identify retention times and exclude lipid species present at low levels and/or not detected consistently. The final optimized method was used to analyze lipids in experimental samples, with data collected for three technical replicates of each sample. Data were analyzed using AB Sciex MultiQuant software (Framingham, MA, USA) for peak finding and integration. The raw peak areas were normalized for recovery of the appropriate internal standards. Lipid species with coefficients of variation greater than 20% were excluded from the final report.

### 2.4. Lipidomics Analysis

The LipidSig differential analysis tool was used to analyze the lipidomics data using the web-based platform [27]. The lipidomics data were normalized to the sample weight, and duplicate values were removed before analysis (*n* = 5 for VEH and *n* = 6 for PEG-BR). The normalized data and sample annotations were uploaded to LipidSig for analysis. Lipid species with more than 50% missing samples were excluded from the analysis. A t-test method was used to identify different species. For global visualization of altered lipid species, hierarchical clustering was performed with a Pearson distance measure and a complete clustering method. Only significantly changed lipid species were analyzed using hierarchical clustering. Differential lipid species were shown using a relative signal intensity range and visualized via a heatmap. BioPAN on Lipid Maps was used to create the network analysis via the web-based platform [28]. Input data files can be found in the project’s GitHub repository (https://github.com/The-Hinds-Lab/Pegylated-Bilirubin-Nanoparticles-Lipidomics, accessed on 23 December 2022).

To ensure the rigor of the lipidomics analysis, we performed a second analysis. This time the data was normalized to the median abundance of lipid species. After quality control and assurance, the data was log-transformed. If more than 3 were missing for a given lipid species, missing values were imputed. If there were fewer than three values, all values were replaced with the imputed value above. For this analysis, we had an *n* = 4 for both groups. A *t*-test was used to analyze the log2-transformed data for statistical significance. *p*-values were adjusted using the Benjamini–Hochberg method. We then performed binomial enrichment, where each lipid formula is broken down into lipid class, total carbon chain length, the total number of double bonds, single chain lengths, and single chain double bonds. From these, we group the lipids with shared annotations. Then, for each annotation, we counted how many lipids had positive log-fold-changes and negative log-fold-changes (regardless of the adjusted *p*-value) and compared the positive LFC/negative LFC to an expected random ratio of 0.5 using a binomial test.

### 2.5. Quantitative Real-Time PCR Analysis

The measurement of gene expression via real-time PCR was performed as previously described [18]. Total RNA was harvested from the VEH- and PEG-BR-treated mouse livers. Briefly, the tissue was homogenized using a Qiagen Tissue Lyser LT (Qiagen, Hilden, Germany) and then extracted using phenol-chloroform extraction followed by purification using the RNeasy Mini Kit (Qiagen). Total RNA concentration was read on a NanoDrop 2000 spectrophotometer (Thermo Fisher Scientific, Wilmington, DE, USA), and cDNA was synthesized using a High-Capacity cDNA Reverse Transcription Kit (Applied Biosystems, Waltham, MA, USA). PCR amplification of the cDNA was performed by quantitative real-time PCR using TrueAmp SYBR Green qPCR SuperMix (Alkali Scientific, Fort Lauderdale, FL, USA) for gene-specific primers (Table 1) and as previously described [17,21,29]. The thermocycling protocol consisted of 5 min at 95 °C, 40 cycles of 15 s at 95 °C, and 30 s at 60 °C, finishing with a melting curve ranging from 60 to 95 °C to allow distinction of specific products. Normalization was performed in separate reactions with primers to 36B4.

### 2.6. Statistical Analysis

The real-time PCR data were graphed and analyzed using Prism 9 (GraphPad Software, San Diego, CA, USA) using an analysis of variance and Tukey’s post-hoc test compared to the group means. A two-tailed, unpaired t-test was used to determine statistical significance when comparing two groups. *p*-values of 0.05 or smaller were considered statistically significant.

## 3. Results

### 3.1. Lipid Family Clustering

**Figure 1:**
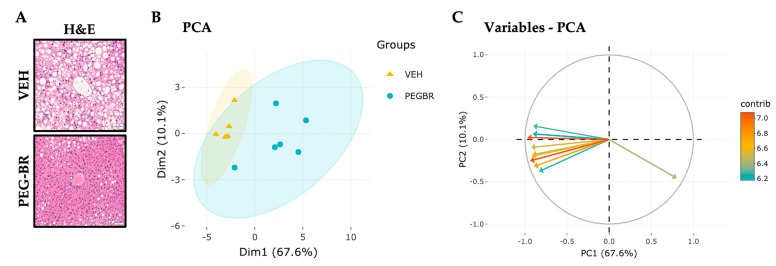
**Hepatic lipids and lipid species clusters in the livers of obese mice treated with bilirubin nanoparticles or vehicle**. (**A**) Hematoxylin and eosin (H&E) staining of bilirubin nanoparticles (PEG-BR) and vehicle (VEH) treated mouse livers (scale bar = 50 μm). (**B**) PCA plot showing the clustering of the VEH and PEG-BR liver lipid species, and (**C**) showing the top lipid species (arrows) and their contribution to the clustering.

To determine how bilirubin affects hepatic lipid species in NAFLD, mice were placed on a HFD for 24 weeks, followed by treatments with VEH or PEG-BR for 4 weeks while remaining on the HFD. The VEH-treated mice developed NAFLD, which was reduced in the PEG-BR-treated animals (Figure 1a). Lipidomics was then performed as previously described in [30], which detected 707 lipid species in both groups. After normalization and quality control, 693 lipid species were used for the subsequent analyses. The PCA plot in Figure 1b was used to visualize the overall clustering of the data, and two distinct groups were visualized between the treatments. Figure 1c shows the 10 top lipid species and their contributions to the clustering observed in Figure 1b. The red arrows denote the lipid species that contributed to the clustering, and the blue arrows indicate a lesser role. Most arrows point to the left, denoting a decrease in lipid species in the PEG-BR-treated group versus the vehicle-treated group. Figure 1 shows the overall changes in the hepatic lipids and lipidomics data by PEG-BR, and next, the specific lipid species were identified.

### 3.2. Significantly Changed Lipid Species

**Figure 2:**
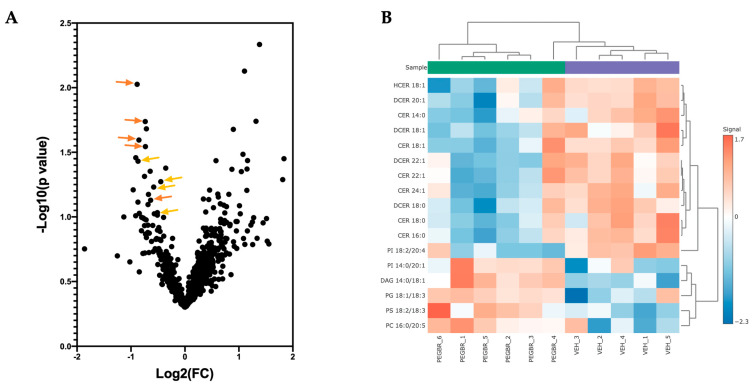
**Bilirubin nanoparticles reduce the number of ceramides and dihydroceramides in the liver of obese mice.** (**A**) A volcano plot showing all lipid species measured. The orange arrows point to ceramides, and the yellowish-orange arrows point to dihydroceramides. (**B**) A heatmap showing the most significantly different lipid species in the PEG-BR vs. VEH groups.

**Figure 3:**
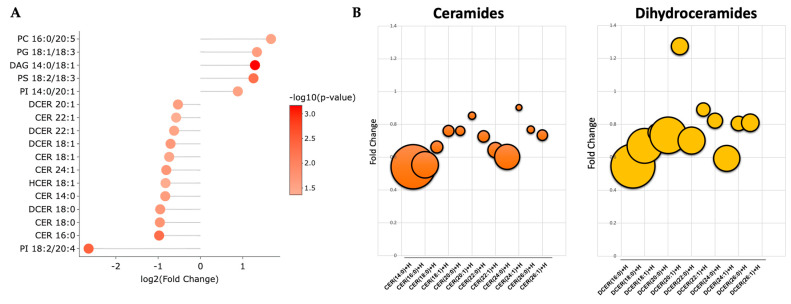
**Bilirubin nanoparticles inhibit the accumulation of ceramides within the liver of obese mice.** (**A**) Shows the log2(fold change) for the statistically significant lipid species. The color of the bubbles denotes the −log10(*p*-value), with the darker shades of red indicating greater significance. (**B**) Bubble plots of the ceramide and dihydroceramide lipid species with different chain lengths. The size of the bubble denotes the −log10(*p*-value), with the larger bubbles indicating more significance.

A volcano plot analysis was used to compare the overall changes in lipid species (Figure 2a). The volcano plot shows the changes in individual lipid species by PEG-BR compared to VEH that were measured. We determined that there was a cluster of ceramides (orange) and dihydroceramides (yellow), as indicated by the lower arrows in the PEG-BR-treated group. We then determined the identity of the significantly changed lipid species. In Figure 2b, we show that the lipid species were significantly different, with five increasing and twelve decreasing. The PEG-BR treatments most significantly reduced phosphoinositide (PI) 18:2/20:4 in the liver. Of the lipid species that were significantly decreased, 11 belonged to the ceramide class, including ceramides (CER), dihydroceramides (DCER), and hexosyl-ceramides (HCER) (Figure 3a). To get a better look at the different ceramide and dihydroceramide species, we created a bubble plot of the log fold change (y-axis) and −log10(*p*-value) (bubble size) for each ceramide and dihydroceramide species measured (Figure 3b). We observed a greater change in the shorter chain length fatty acids (CER 16:0, CER 18:0, DCER 18:0, and CER 14:0).

### 3.3. Validation of Significantly Changed Lipid Species

**Figure 4:**
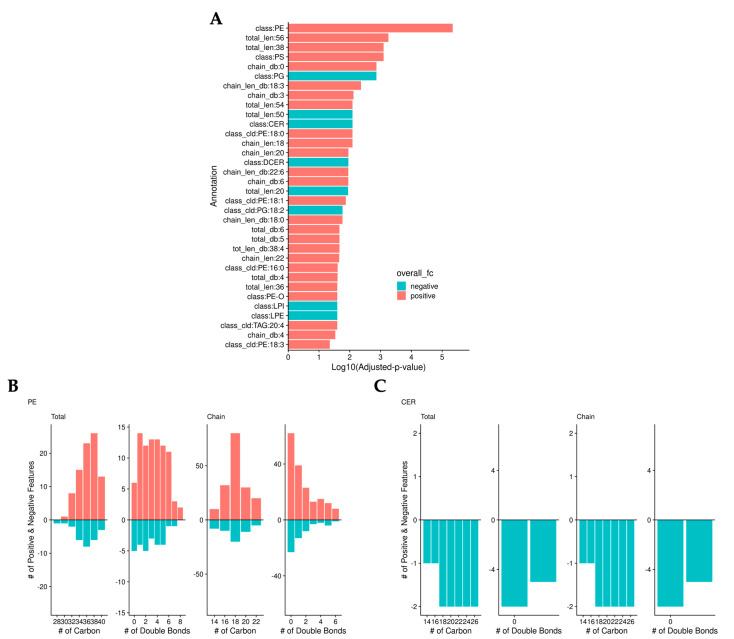
**Bilirubin nanoparticles regulate phosphatidylethanolamine (PE) and ceramide (CER) levels within the liver of obese mice.** (**A**) Log_10_(adjusted-p-values) from binomial tests of lipid annotations. Color represents whether there are more positive (red) or negative (blue) log-fold-change lipids. The number of positive (red) and negative (blue) LogFC lipids is determined by the total and chain numbers of carbon and double bonds in PE (**B**) and CER (**C**).

To validate the previous findings, we normalized the data by median abundance instead of sample mass and calculated adjusted p-values. As we found in the previous analysis, bilirubin lowered the level of ceramides. However, this analysis identified that bilirubin nanoparticles also significantly increased the levels of phosphatidylethanolamine (PE) (Figure 4a). Bilirubin nanoparticles increased the level of most PEs regardless of the number of carbons or double bonds (Figure 4b). Bilirubin nanoparticles lowered the amount of all ceramide lipid species (Figure 4c).

### 3.4. Network Analysis

**Figure 5:**
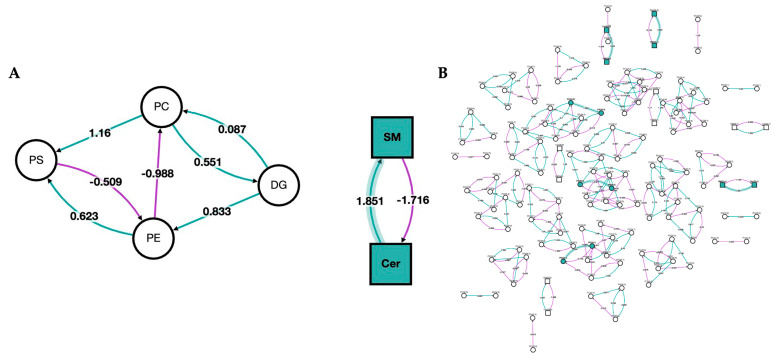
**Network of lipid classes and species changed in bilirubin nanoparticles and vehicle-treated obese mice.** Network analysis of PEG-BR vs. VEH-treated mouse livers. (**A**) Network analysis of the lipid class; and (**B**) network analysis of the lipid species. Node shape denotes the lipid type, with circles representing glycerolipids and glycerophospholipids and squares representing sphingolipids. The node color represents whether the lipid class or species was changed by the PEG-BR (green) or remain unchanged (white). The purple line denotes a negative z-score, and the green line indicates a positive z-score. The shaded line indicates that the z-score is implied in a lipid class or species that was changed by PEG-BR.

In Figure 5, we show a network analysis of the lipid classes and species in the bilirubin nanoparticles and vehicle-treated obese mice. The green color represents the classes/species that are changed by the bilirubin nanoparticle treatments. The network analysis shows that the PEG-BR primarily altered sphingomyelins (SM) and ceramides (Cer).

### 3.5. RTPCR of the Ceramide Synthesis Pathway

To determine whether the bilirubin nanoparticle compared to the vehicle-treated obese mice had changes in the mRNA expression of key enzymes that regulate ceramide production, we used real-time PCR to measure the expression of genes within the ceramide synthesis pathways in the livers (Table 2). The bilirubin nanoparticle treatment did not significantly change the expression of ceramide synthases (*Cers1–6)*. However, they did significantly reduce the expression of *Sgpl1* (*p* = 0.0092) and *Degs1* (*p* = 0.0283), which are enzymes involved in generating fatty acids [14,31,32].

## 4. Discussion

Plasma bilirubin levels are negatively associated with obesity in humans [33] and rodents [6,34,35]. Several studies have demonstrated that hepatic bilirubin deficiency promotes steatosis [17,36,37,38], and treatment with bilirubin to raise plasma levels reverses dietary-induced hepatic fat accumulation [7,11,17,38,39,40]. However, the effect of bilirubin on hepatic fat metabolism and lipid species has not been previously studied in detail. The current study demonstrated for the first time that bilirubin nanoparticles modified the hepatic lipid pool by increasing the amount of phosphatidylethanolamine and inhibiting the accumulation of ceramides within the liver. The reduction in hepatic ceramides by bilirubin nanoparticles in this study could be a potential mechanism by which bilirubin significantly reduces insulin resistance in mice on a high-fat diet [10,41]. The accumulation of ceramides has been shown to be associated with insulin resistance by inhibiting Akt, leading to a decrease in GLUT4-mediated glucose uptake [42]. Ceramides activate atypical protein kinase C zeta (PKCζ), which phosphorylates Akt at an inhibitory site in the PH domain [43]. Ceramides also mediate lipotoxicity by promoting endoplasmic reticulum (ER) stress and inflammation and by activating caspases that result in proteolysis, nuclear fragmentation, and apoptotic cell death [12].

As a result of our analysis in this study, we also observed that bilirubin nanoparticle treatment significantly increased the amount of phosphatidylethanolamine in the liver of obese mice. Prior studies have identified that a lower ratio of phosphatidylcholine to phosphatidylethanolamine is associated with developing NAFLD [44]. By inhibiting ER stress and reducing reactive oxygen species (ROS), phosphatidylethanolamine can protect against the development of NAFLD [45]. Likewise, bilirubin treatments in leptin receptor-deficient *db/db* mice were demonstrated to enhance insulin sensitivity through inhibition of ER stress [46]. ROS can contribute to the pathogenesis of NAFLD, and it has been proposed that targeting ROS through heme oxygenase-1-mediated bilirubin production could be a potential therapeutic for NAFLD [39]. Bilirubin decreases ROS production through direct ROS scavenging and by inhibiting NADPH oxidase, which is the enzyme responsible for ROS production [47,48,49,50,51].

In this study, we show that bilirubin nanoparticle treatments in obese mice inhibited the accumulation of ceramides and suppressed the hepatic expression of *Sgpl1* and *Degs1.* Ruangsiriluk et al. demonstrated that silencing enzymes such as *Degs1*, which are involved in ceramide biosynthesis, causes distinct global alterations of lipid homeostasis and gene expression [31]. Chaurasia et al. showed that targeting *Degs1* improved insulin resistance and hepatic steatosis [52]. Similarly, suppression of *Sgpl1* disrupts lipid homeostasis in the liver and reduces total body fat [32]. The *Sgpl1* gene produces sphingolipids, which have been shown to induce inflammation [53]. Not surprisingly, bilirubin and bilirubin nanoparticles have been shown to reduce inflammation [11,41,46].

There are a few limits to the current study. First, previous studies show that roughly 95% of the transcriptional responses to bilirubin in human hepatocytes are mediated by PPARα [9]. However, it is unknown whether the changes in lipid species and the decreased mRNA expression in this study are mediated by PPARα. Follow-up studies are needed using PPARα knockout mice to determine if bilirubin causes these changes through a PPARα-dependent mechanism. Secondly, while the HFD model utilized in the present study causes significant steatosis and inflammation, which are alleviated by bilirubin nanoparticle treatment, this model does not develop significant hepatic fibrosis, so the effect of bilirubin nanoparticle treatment on fibrosis is not known. The effect on fibrosis can be studied in other mouse models of NASH [18].

In conclusion, we show that bilirubin nanoparticle treatments remodeled the liver fat content by reducing ceramides and increasing phosphatidylethanolamine, which promotes metabolic health. The bilirubin nanoparticles might be useful for regulating disorders where ceramide levels are elevated, particularly those observed in metabolic diseases such as fatty liver disease. There are other incidences where ceramides are elevated and bilirubin nanoparticle treatment might be useful, such as obesity, diabetes, cancer, hepatic steatosis, NAFLD, hypertension, heart failure, and atherosclerosis. These are supported by our work and that of Ai et al., who demonstrated that bilirubin nanoparticles improve cardiovascular phenotypes in mice with ischemia/reperfusion (I/R) injury [54]. Future work to further reveal the mechanistic actions of the bilirubin nanoparticles and their safety is needed. Studies have shown that bilirubin nanoparticles improve liver function as measured by significantly reduced levels of the liver dysfunction biomarker, AST, and lower hepatic inflammation [11]. Other factors to investigate include the duration of treatments to reduce ceramide production and how long the bilirubin nanoparticle treatments sustain reduced levels for protection. An additional consideration in future studies is how the bilirubin nanoparticles might improve cardiovascular diseases through a reduction of ceramides. Future studies to determine the roles of bilirubin in other, less-known tissues, such as muscle or kidney, are also needed. Overall, bilirubin nanoparticles show therapeutic potential in metabolic and cardiovascular diseases.

## Figures and Tables

**Table 1:** Sequences of primers used in this study.

Gene	Forward Primer	Reverse Primer
*Cers1*	AGCGGAGACAGCGGAGAAT	GCATAACTCGGCATGGGCTC
*Cers2*	GCTAGAAGTGGGAAACGGAGT	GCCATAGTCGTTCCCACCAG
*Cers3*	TCCAGTAGCTTTGCCTCACG	CGTTCCAGGCAGCTTTGTTC
*Cers4*	GGTCTGCGGCTTGTCTAAGG	CTGAACGACATGCTGGAGGT
*Cers5*	TTCGCCATCGGAGGAATCAG	AACCAAGGCATCGACCAGAG
*Cers6*	CGGAGCCTGAGAGTGCAG	CCTGCCATCTTGCTTTGTCC
*Sgpl1*	TCTGCGGGGAAAGAAGGAGC	GCTTGAGGAGGTCGGTTCC
*Sgppl1*	AATCTCGACCCCTTTGTGGG	CAGCGGATGATGTCCTTGGT
*Sptch1*	GGAGTCACCGAGCACTATGG	AGGGGAGGTAACGAAGCAGA
*Kdsr*	CAAAACGAAGCCCCTGGAGA	AGCATGTACCCATCTGAGCC
*Smpd1*	GTGGGACTCCTTTGGATGGG	CCCAAAGAACCGTGGAGTCA
*Sgms1*	TATGGGTGGACACTTGGGCT	AGCCTGTGTGGTCTATGGTG
*Sphk1*	TCCTGGGCAACACCGATAAG	ACTGGTTCCATAGCCAGGTC
*Sphk2*	ATGATCGGAGCTTGCTGGAC	GCCAGGCCAAGTGTTGAAAG
*Asah1*	AGCAGGTTTGAAACGCCAGAG	TAATTCTCACCCCCGACTCCT
*Degs1*	AATGGGTCTACACGGACCAG	GGACGAGAAGCATCATGGCTA
*Cerk*	CTGACTGGGAGCACTGACAC	GGATGAGGGGAGGCCATAGT
*Ugcg*	GTGTGACGGGGATGTCTTGT	GAAAACCTCCAACCTCGGTC
*Galc*	ACCCGCACAATGGCTAACA	ACAACAATAAGGGCACCGCA
*Ptdss1*	CTCCGGGTCACCGATACCTA	CGTATCCCCGGCGTAGGTTG
*Ptdss2*	CGTCCCACAATGCCTCACG	TGAGCAGCGGAGACTCAGA
*Pemt*	GGCAATATCGACTTCAGGCAGG	CCATCTCGCTACCACATTCCA

**Table 2:** Fold Change (FC) of real-time PCR expression of genes in the livers of obese mice treated with PEG-BR or VEH.

Gene	FC PEG-BR vs. VEH	*p*-Value
*Cers1*	1.06	0.788
*Cers2*	0.93	0.433
*Cers3*	1.21	0.532
*Cers4*	0.945	0.621
*Cers5*	0.82	0.132
*Cers6*	0.81	0.296
*Sgpl1*	0.78	0.0092 *
*Sgppl1*	1.05	0.754
*Sptch1*	0.89	0.213
*Kdsr*	0.80	0.114
*Smpd1*	0.96	0.439
*Sgms1*	0.86	0.500
*Sphk1*	0.98	0.929
*Sphk2*	0.90	0.320
*Asah1*	1.05	0.871
*Degs1*	0.83	0.0283 *
*Cerk*	1.13	0.456
*Ugcg*	0.97	0.74
*Galc*	0.88	0.321
*Ptdss1*	0.91	0.321
*Ptdss2*	0.87	0.47
*Pemt*	0.85	0.105

* denotes *p* < 0.05.

## Data Availability

All data and supplemental information regarding this manuscript are included on GitHub or can be provided upon request.
